# The impact of Typhoon Haiyan on admissions in two hospitals in Eastern Visayas, Philippines

**DOI:** 10.1371/journal.pone.0191516

**Published:** 2018-01-30

**Authors:** Joris Adriaan Frank van Loenhout, Julita Gil Cuesta, Jason Echavez Abello, Juan Mari Isiderio, Maria Lourdes de Lara-Banquesio, Debarati Guha-Sapir

**Affiliations:** 1 Centre for Research on the Epidemiology of Disasters (CRED), Institute of Health and Society, Université catholique de Louvain, Brussels, Belgium; 2 Eastern Visayas Regional Medical Center, Tacloban, Philippines; 3 Ormoc District Hospital, Ormoc, Philippines; Universidade Nova de Lisboa Instituto de Higiene e Medicina Tropical, PORTUGAL

## Abstract

**Objectives:**

We investigated the short-term impact of Typhoon Haiyan, one of the strongest typhoons ever to make landfall, on the pattern of admissions in two hospitals in Eastern Visayas, the Philippines.

**Methods:**

This study took place at Eastern Visayas Regional Medical Center (EVRMC) in Tacloban, and Ormoc District Hospital (ODH) in Ormoc. We determined whether there were differences in the pattern of admissions between the week before and the three weeks after Haiyan by using information on sex, age, diagnosis, ward and outcome at discharge from patient records.

**Results:**

There was a drop in admissions in both hospitals after Haiyan as compared to before. Admissions climbed back to the baseline after ten days in EVRMC and after two weeks in ODH. When comparing the period after Haiyan to the period before, there was a relative increase in male versus female admissions in ODH (OR 2.8, 95%CI 1.7–4.3), but not in EVRMC. Patients aged ≥50 years and 0–14 years had the highest relative increase in admissions. There was a relative decrease in admissions for the ICD10 group ‘Pregnancy, childbirth and the puerperium’ (OR 0.4, 95%CI 0.3–0.6), and an increase in ‘Certain infectious and parasitic diseases’ (OR 2.1, 95%CI 1.2–3.5), mainly gastroenteritis, and ‘Diseases of the respiratory system’ (OR 1.8, 95%CI 1.0–3.0), mainly pneumonia, compared to all other diagnosis groups in ODH. Out of all reasons for admission within the study period, 66% belong to these three ICD-10 groups. Data on reasons for admission were not available for EVRMC.

**Conclusions:**

The observed reduction in patients after the Typhoon calls for ensuring that hospital accessibility should be protected and reinforced, especially for pregnant women, by trying to remove debris in the direct hospital vicinity. Hospitals in areas prone to tropical cyclones should be prepared to treat large numbers of patients with gastroenteritis and pneumonia, as part of their disaster plans.

## Introduction

Tropical cyclones are called typhoons if they occur in the northwestern Pacific Ocean and hurricanes in the Atlantic and northeastern Pacific Ocean. Damage due to this type of event occurs because of strong winds, which in turn can lead to storm surges, a tsunami-like phenomenon. These types of events can have a large impact on the number of hospital admissions, as well as on the cause for hospitalisation, as has been shown in previous studies. The number of admissions within six hospitals in Hampton Roads, Virginia (USA), increased on the day of Hurricane Isabel, but recovered to the baseline level the day after [1]. However, trauma admissions were still elevated two and three days after the event. Patients who missed at least one dialysis session during Hurricane Katrina in New Orleans (nearly 50%, and more often women) had much higher odds of being hospitalised in the following month [2]. An increase in the incidence of pneumonia hospitalisations among adults was found after an earthquake and the following tsunami in 2011 in northern Miyagi, Japan [3].

There is very limited research on the impact of tropical cyclones on hospital admissions in low- and middle-income countries. Patient data collected from a field hospital in Banda Aceh, Indonesia, after the 2004 tsunami showed no increase in cholera, malaria and dengue in the four months following the event [4]. However, there was an increase in the number of wounds and injuries, tetanus cases and respiratory infections, especially in the first six weeks after the tsunami [4]. Less than one third of patients with respiratory infections consisted of children under the age of five years.

**Fig 1:**
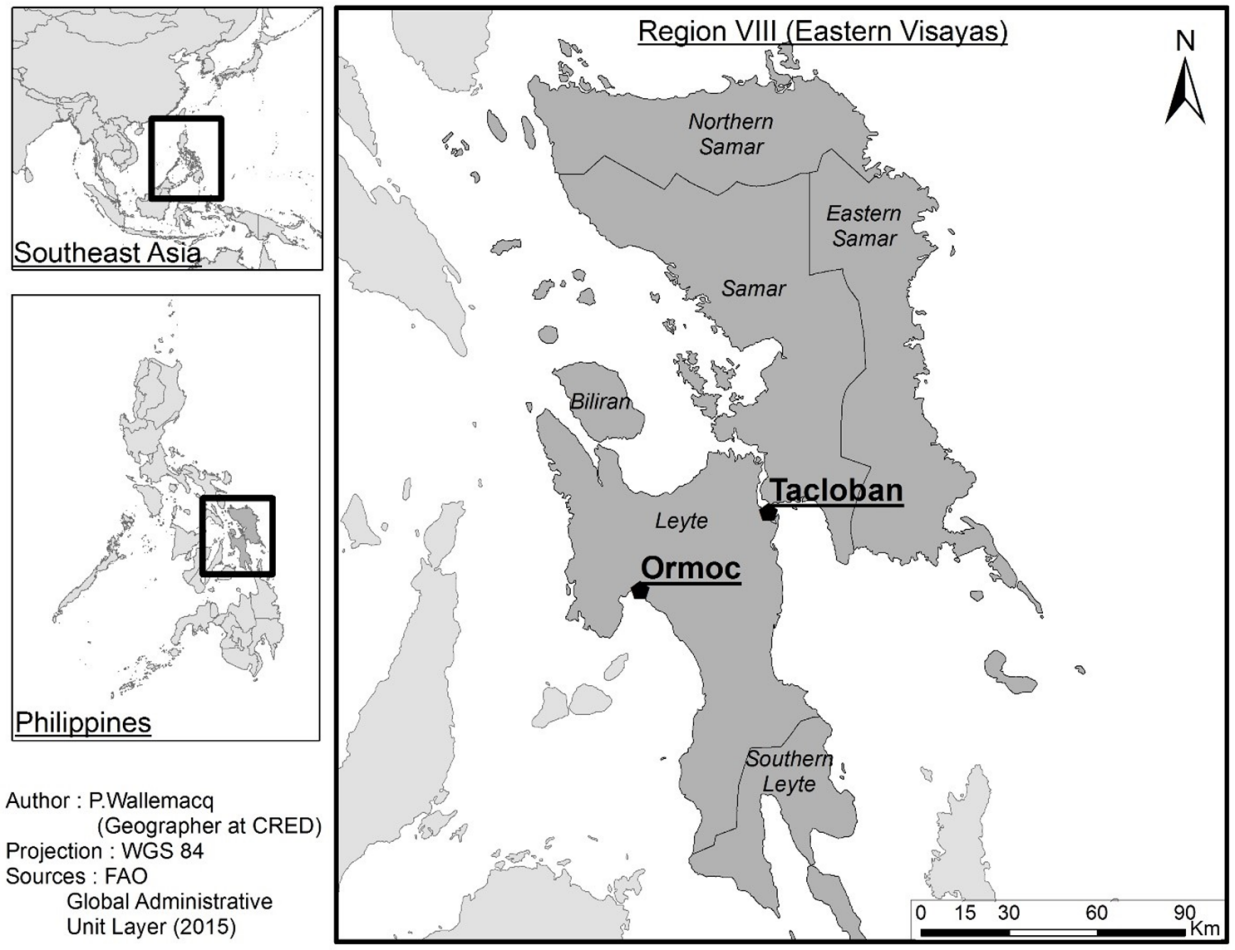
Representation of Eastern Visayas, the area affected by Typhoon Haiyan. The study locations are indicated on the map (Ormoc District Hospital in Ormoc, and Eastern Visayas Regional Medical Center in Tacloban).

On the 8^th^ of November 2013, the Philippines was hit by Typhoon Haiyan, which was the strongest typhoon ever to make landfall on a major island in the western North Pacific Ocean [5]. The impact was enormous, with more than 7,000 deaths and 16 million people affected [6]. The city of Tacloban in the region Eastern Visayas (Fig 1) was hit hardest by the occurrence of a storm surge of up to 5 meters [5], due to which many people drowned, especially people aged 55 years and older and those who lived close to the sea [7]. In the first month after Haiyan, 108 foreign medical teams were sent to the Philippines [8]. Field hospitals were set up to support local hospitals with the diagnosis and treatment of communicable [9] and chronic diseases [10], and performing surgeries [11].

This study investigated the short-term impact of Typhoon Haiyan on admissions in two hospitals that remained operational during and after the typhoon in Eastern Visayas, Philippines. We examined whether there were differences in the pattern of admissions between the week before and the three weeks after Haiyan in terms of age, sex, the ward in which patients were admitted, their diagnosis and their outcome at discharge. The general assumption is that women and children are mostly affected by disasters, but until now there is almost no evidence to back this up. The results of this study help to fill the existing gap on the impact of tropical cyclones on hospital admissions in low- and middle-income countries, and it is essential for preparation of hospitals for future events.

## Methods

### Study setting

The study was carried out in the region of the Philippines that was hardest hit by the typhoon, the Eastern Visayas. Fig 1 shows this area, including the location of the two study sites. Data collection took place in two public hospitals: *Eastern Visayas Regional Medical Center* (EVRMC), a tertiary hospital in Tacloban, and *Ormoc District Hospital* (ODH), a secondary hospital in Ormoc. The capacities of EVRMC and ODH in 2013 were 250 and 100 beds, respectively. In EVRMC, there was an operational disaster plan in place, which was not the case for ODH. Although both hospitals remained operational, they were partly damaged by the typhoon. EVRMC was hit by strong winds as well as a storm surge, while ODH only experienced strong winds [5]. In both sites, there was very limited electricity during several days, parts of the infrastructure were destroyed, and the availability of medical and other supplies was affected. Staff had difficulty reaching the hospital, due to debris on the roads [12]. Despite these limitations, both hospitals did not stop admitting patients [13].

### Data collection

Data from patient records were collected from centralised hospital registries. Patient records were summarised in logbooks in EVRMC and in Excel files in ODH. The research team collected data on all admissions that took place in November 2013, one week before (baseline) and approximately three weeks after Typhoon Haiyan. Admission date, sex, age, ward in which the patient was admitted, final diagnosis and patient outcome at discharge were available from patient records. In EVRMC, the research team entered patient data from logbooks in September 2016, using the software EpiData 3.1.

### Data description

We grouped age in four categories: 0–4, 5–14, 15–49 and ≥50 years old. The research team reclassified all diagnoses according to the International Classification of Diseases, ICD-10 version 2016, up until the third level [14]. If patients had multiple diagnoses, we categorised them under multiple ICD-10 codes. The patient outcome at discharge was regrouped from ‘Improved’, ‘Recovered’, ‘Discharged’ and ‘Discharged against medical advice’ into ‘Alive’, and from ‘Died’ into ‘Death’.

### Data analysis

We made a descriptive overview of the study variables for the baseline week (November 1–7), and each week after Typhoon Haiyan (November 8–14, 15–21, 22–28). We carried out logistic regression, comparing variables between the baseline period before Typhoon Haiyan (November 1–7) and the period after (November 8–30). As Haiyan occurred in the early morning of November 8, we included the 8^th^ in the period after the typhoon. The hospitals were located on different sides of the island, and they were affected by different weather phenomena. EVRMC is a tertiary hospital and ODH secondary, so the number and complexity of cases they receive is different. For the above reasons, we decided to analyse and present the results separately.

For EVRMC, data on diagnosis and patient outcome at discharge were available for only 25% of the cases. Therefore, we decided not to include these variables for the EVRMC analyses, but instead we used ward as a proxy for diagnosis (e.g. Obstetrics ward for diagnoses related to pregnancy and childbirth). All other variables had few missing values for both hospitals (>90% complete).

### Ethical considerations

Patient data were used anonymously. Ethical clearance was waived by the Institutional Ethics Review Committee of EVRMC, since it was a retrospective study using chart records.

## Results

**Fig 2:**
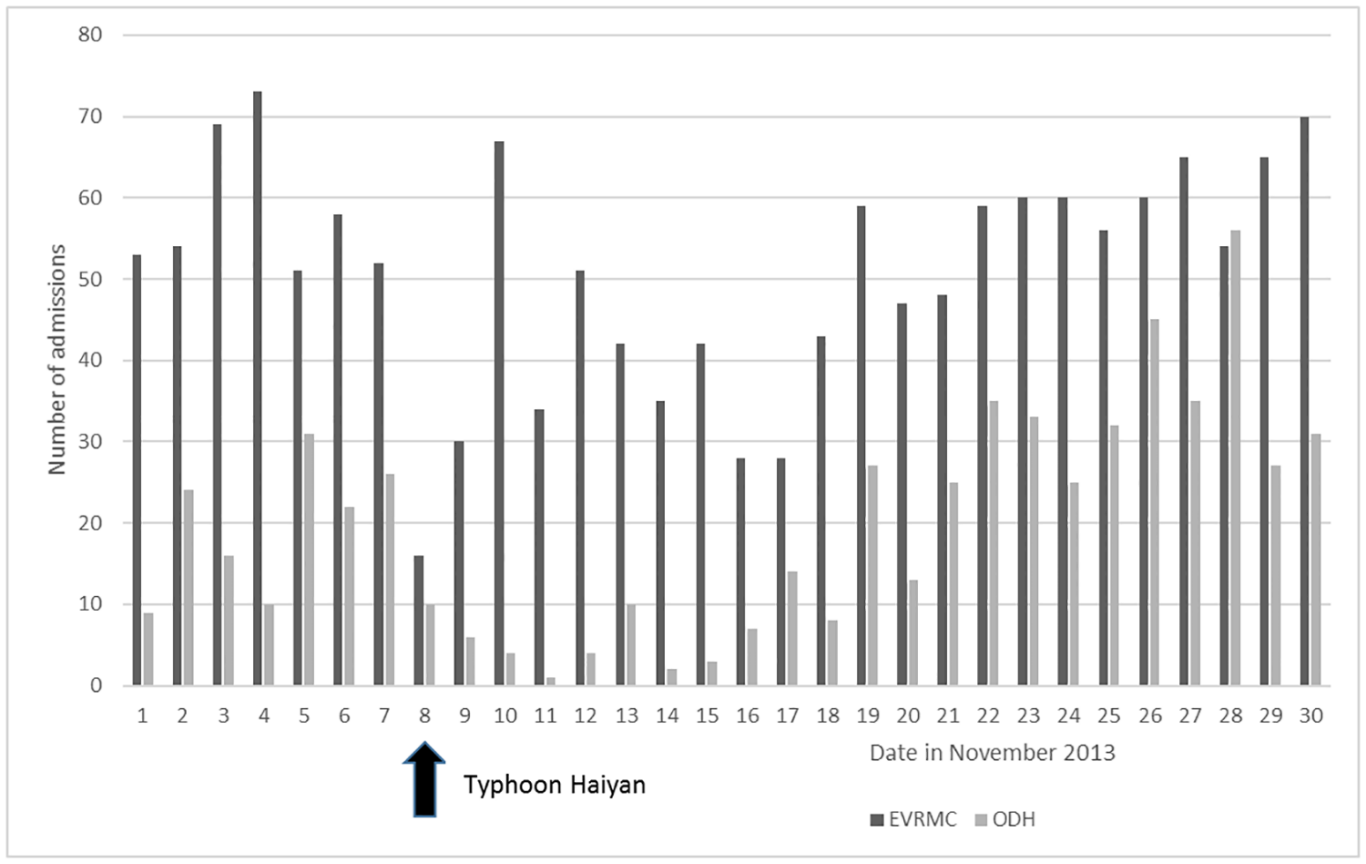
Number of daily hospital admissions before and after Typhoon Haiyan (November 2013) for Eastern Visayas Regional Medical Center (EVRMC) and Ormoc District Hospital (ODH).

The daily number of admissions before and after Haiyan for both hospitals is shown in Fig 2. On the day of Typhoon Haiyan (November 8), there was a drop in admissions in both hospitals. Admissions in EVRMC climbed back to the baseline after ten days. In contrast, while admissions in ODH had also dropped significantly after the typhoon, they increased steadily and surpassed the baseline two weeks after the event.

### Characteristics of admissions over time

The numbers of admissions per week in terms of sex, age, ward, patient outcome at discharge and diagnosis are presented in Tables 1 and 2. In both hospitals, the overall proportion of women was higher compared to men, although the proportion of women dropped in ODH after Haiyan. The most frequently admitted age groups were 0–4 and 15–49 years. In both hospitals, the Obstetrics ward admits the largest share of all inpatients during the complete study period. However, in the post-typhoon period, the Paediatrics and Medicine wards claimed a larger share. In ODH, the most common diagnoses over the whole study period were for the ICD10 groups ‘Pregnancy, childbirth and the puerperium’ (mainly ‘O80, Single spontaneous delivery’, n = 156), ‘Certain infectious and parasitic diseases’ (mainly ‘A09, Other gastroenteritis and colitis of infectious and unspecified origin’, n = 101) and ‘Diseases of the respiratory system’ (mainly ‘J18, Pneumonia, organism unspecified’, n = 104) (Table 2). The share of deliveries among the ICD10 group ‘Pregnancy, childbirth and the puerperium’ was 94%. The overall proportion of deaths among admitted patients in ODH was 2.8%.

**Table 1:** Weekly admissions in Eastern Visayas Regional Medical Center in terms of sex, age and ward, one week before and three weeks after Typhoon Haiyan.

		Week -1 (baseline)		Week 1 (post-typhoon)		Week 2		Week 3		Total	
		n	%	n	%	n	%	n	%	n	%
Total admissions		410	29.4	275	19.7	295	21.2	414	29.7	1394	100
Sex											
	Male	175	42.8	102	37.1	121	41.2	193	46.7	591	42.5
	Female	234	57.2	173	62.9	173	58.8	220	53.3	800	57.5
Age											
	0–4 years	139	34	95	34.7	98	33.3	130	31.6	462	33.3
	5–14 years	46	11.2	21	7.7	27	9.2	32	7.8	126	9.1
	15–49 years	161	39.4	130	47.4	113	38.4	153	37.1	557	40.1
	≥50 years	63	15.4	28	10.2	56	19	97	23.5	244	17.6
Ward											
	Obstetrics	104	25.4	86	31.4	85	28.8	81	19.6	356	25.6
	NICU	84	20.5	70	25.5	69	23.4	65	15.7	288	20.7
	Medicine	71	17.3	25	9.1	49	16.6	111	26.8	256	18.4
	Pediatrics	63	15.4	35	12.8	48	16.3	86	20.8	232	16.7
	Surgery	72	17.6	37	13.5	32	10.8	35	8.5	176	12.6
	Orthopedics	7	1.7	18	6.6	8	2.7	19	4.6	52	3.7
	Isolation	7	1.7	2	0.7	2	0.7	7	1.7	18	1.3
	EENT	2	0.5	1	0.4	0	0	6	1.4	9	0.6
	ICU	0	0	0	0	2	0.7	4	0.9	6	0.5

NICU = Neonatal Intensive Care Unit; EENT = Eye, Ear, Nose and Throat; ICU = Intensive Care Unit

**Table 2:** Weekly admissions in Ormoc District Hospital in terms of sex, age, ward, patient outcome at discharge and diagnosis, one week before and three weeks after Typhoon Haiyan.

		Week -1 (baseline)		Week 1 (post-typhoon)		Week 2		Week 3		Total	
		n	%	n	%	n	%	n	%	n	%
Total admissions		138	25.9	37	6.9	97	18.2	261	49	533	100
Sex											
	Male	27	19.6	16	43.2	44	45.4	96	36.8	183	34.3
	Female	111	80.4	21	56.8	53	54.6	165	63.2	350	65.7
Age											
	0–4 years	18	13.3	6	20.0	31	32.6	60	23.5	115	22.3
	5–14 years	8	5.9	2	6.7	10	10.5	19	7.5	39	7.6
	15–49 years	86	63.7	15	50.0	42	44.2	137	53.7	280	54.4
	≥50 years	23	17.0	7	23.3	12	12.6	39	15.3	81	15.7
Ward											
	Obstetrics	71	51.4	12	33.3	19	19.6	79	30.5	181	34.2
	Paediatrics	19	13.8	7	19.4	39	40.2	70	27.0	135	25.5
	Medicine	32	23.2	6	16.7	29	29.9	64	24.7	131	24.7
	Surgery	11	8.0	11	30.6	9	9.3	41	15.8	72	13.6
	Gynaecology	5	3.6	0	0.0	1	1.0	4	1.5	10	1.9
	Orthopaedics	0	0.0	0	0.0	0	0.0	1	0.4	1	0.2
Patient outcome											
	Alive	131	95.6	35	97.2	95	99.0	253	97.3	514	97.2
	Death	6	4.4	1	2.8	1	1.0	7	2.7	15	2.8
ICD10 Group^1^											
	Pregnancy	65	51.6	9	30.0	21	22.3	82	32.3	177	35.1
	Infections	19	15.1	10	33.3	28	29.8	62	24.4	119	23.6
	Respiratory	18	14.3	4	13.3	27	28.7	56	22.0	105	20.8
	Genitourinary	9	7.1	1	3.3	9	9.6	23	9.1	42	8.3
	Digestive	8	6.3	0	0.0	5	5.3	23	9.1	36	7.1
	Circulatory	9	7.1	2	6.7	5	5.3	18	7.1	34	6.7
	Injury	5	4.0	5	16.7	7	7.4	17	6.7	34	6.7

^1)^ Only ICD10 groups with more than 20 admissions in November 2013 are shown. Proportions do not add up to 100%, since patients could be admitted for more than one diagnosis. Group names are abbreviated from ‘Pregnancy, childbirth and the puerperium’, ‘Certain infectious and parasitic diseases’, ‘Diseases of the respiratory system’, ‘Diseases of the genitourinary system’, ‘Diseases of the digestive system’, ‘Diseases of the circulatory system’, and ‘Injury, poisoning and certain other consequences of external causes’, respectively.

### Admissions before and after Haiyan

We tested whether there was a proportional increase in admission characteristics in the period after Haiyan (November 8–30) compared to baseline (November 1–7). Table 3 shows there was an increase in male versus female admissions in ODH (OR 2.8, 95%CI 1.7–4.4). This effect was not present for EVRMC. Older age groups (≥50 years) had the largest relative increase after Haiyan in EVRMC. In contrast, the very young (0–4 years) presented an increase in ODH. In EVRMC, there was a relative increase in patients admitted to the Orthopaedics ward (OR 2.5, 95%CI 1.1–5.6) and a decrease for patients in the Surgery ward (OR 0.6, 95%CI 0.4–0.8) compared to all other wards. In ODH after Haiyan compared to before, there was a proportional increase in patients admitted in the Paediatrics ward (OR 2.7, 95%CI 1.6–4.6), and a decrease in the Obstetrics ward (OR 0.4, 95%CI 0.3–0.6) compared to the rest of the wards. In terms of diagnoses, there was a relative decrease in admissions for the ICD10 group ‘Pregnancy, childbirth and the puerperium’ (OR 0.4, 95%CI 0.3–0.6), and an increase in ‘Certain infectious and parasitic diseases’ (OR 2.1, 95%CI 1.2–3.5) and ‘Diseases of the respiratory system’ (OR 1.8, 95%CI 1.0–3.0) compared to all other diagnosis groups. There was no difference in patient outcome at discharge when comparing the baseline and the period after Haiyan in ODH.

**Table 3:** Measures of association for different characteristics, comparing admissions before (November 1–7) and after (November 8–30) Haiyan.

		EVRMC		ODH	
		N = 1529		N = 591	
		OR^3^ (95% CI)	P-value	OR^3^ (95% CI)	P-value
Sex					
	Male	0.96 (0.76 to 1.20)	0.708	2.76 (1.74 to 4.38)	< 0.001
	Female	Ref	Ref	Ref	Ref
Age			0.149		0.011
	0–4 years	0.84 (0.60 to 1.19)	0.323	2.25 (1.13 to 4.46)	0.021
	5–14 years	0.59 (0.38 to 0.94)	0.025	1.44 (0.58 to 3.56)	0.433
	15–49 years	0.88 (0.63 to 1.23)	0.455	0.90 (0.53 to 1.54)	0.699
	≥50 years	Ref	Ref	Ref	Ref
Ward^1^					
	Obstetrics	1.05 (0.81 to 1.36)	0.713	0.38 (0.26 to 0.56)	< 0.001
	Medicine	1.08 (0.80 to 1.45)	0.618	1.12 (0.72 to 1.76)	0.610
	Paediatrics	1.11 (0.81 to 1.51)	0.524	2.71 (1.61 to 4.58)	< 0.001
	Surgery	0.55 (0.40 to 0.76)	< 0.001	1.88 (0.96 to 3.68)	0.065
	NICU	1.05 (0.79 to 1.39)	0.734	N/A	N/A
	Orthopaedics	2.53 (1.13 to 5.64)	0.024	N/A	N/A
	EENT	1.47 (0.31 to 6.95)	0.627	N/A	N/A
	Isolation	0.68 (0.27 to 1.71)	0.410	N/A	N/A
	Gynaecology	N/A	N/A	0.42 (0.13 to 1.35)	0.144
Disposition					
	Alive	N/A	N/A	1.83 (0.66 to 5.04)	0.244
	Death	N/A	N/A	Ref	Ref
ICD10 Group^1^^,^^2^					
	Pregnancy	N/A	N/A	0.41 (0.27 to 0.62)	< 0.001
	Infections	N/A	N/A	2.05 (1.21 to 3.50)	0.008
	Respiratory	N/A	N/A	1.75 (1.01 to 3.03)	0.045
	Genitourinary	N/A	N/A	1.21 (0.57 to 2.58)	0.619
	Digestive	N/A	N/A	1.14 (0.51 to 2.54)	0.758
	Circulatory	N/A	N/A	0.93 (0.43 to 2.02)	0.856
	Injury	N/A	N/A	1.80 (0.68 to 4.73)	0.235

N/A = Not applicable, since data was not available for this analysis or the sample size was too small; Ref = Reference group.

^1)^ For ward and ICD10 group, the category in question was analysed compared to all other categories combined. ORs for the ward ICU could not be calculated, so we did not include it in the table;

^2)^ Only analyses for ICD10 groups with more than 20 admissions in November 2013 are shown. Group names are abbreviated from ‘Pregnancy, childbirth and the puerperium’, ‘Certain infectious and parasitic diseases’, ‘Diseases of the respiratory system’, ‘Diseases of the genitourinary system’, ‘Diseases of the digestive system’, ‘Diseases of the circulatory system’, and ‘Injury, poisoning and certain other consequences of external causes’, respectively;

^3)^ Admissions before Haiyan are the reference group. A value of 2.76 for male vs. female admissions in ODH means that men were 2.76 times as likely to be admitted as women after Haiyan, compared to before the typhoon.

### Sensitivity analysis

The contribution of childbirth-related admissions was large (Tables 1 and 2). Since we expected the number of childbirths to remain relatively stable compared to other reasons for admissions, we tested whether this reason for admission affected our results with respect to sex, age and ward. Therefore, we reanalysed those data after excluding cases with admissions in the Obstetrics ward (n = 396 and 200 for EVRMC and ODH, respectively), and those whose age was stated as new-born (N = 335 and 12 for EVRMC and ODH, respectively). For EVRMC, there were no differences in terms of the direction of associations for age, sex and ward distribution (data not shown). For ODH, the only difference was that within these analyses, admissions within the Gynaecology ward decreased significantly after Haiyan (OR 0.3, 95%CI 0.1–0.9) compared to all other wards.

## Discussion

The results from our study show that there was an overall decrease in the number of admissions in the two weeks after Haiyan. We noted a decrease in admissions for females vs. males in the post-typhoon period. With regard to age groups, the very young and the older populations were more likely to be hospitalised than those between 5–49 years of age after the typhoon. Obstetrics and surgical admissions decreased, in contrast to orthopaedic admissions and hospitalisations for infections and respiratory diseases. The proportion of deaths among admitted patients was low and was not significantly affected by the typhoon.

Typically, acute events such as tropical cyclones and earthquakes do present peaks in the number of admissions [1, 15, 16], although evidence of this is rather sparse, especially on tropical cyclones. In the case of Haiyan, the combined effects of the severity of the typhoon and the ensuing destruction, including road infrastructure, may have played a very important role in the accessibility to the medical facilities in our study. In addition, people might have given lower priority to their medical needs than normal, due to being affected by typhoon-related circumstances such as lack of food and affected housing. The local population may have replaced their needs by medical care from neighbourhood sources or field hospitals, until such time that they were able to access the hospital. A manuscript currently under preparation by the same author group describes the pattern of outpatient consultations in a field hospital operated by the Non-Governmental Organisation Mercy Malaysia in the months following Haiyan. However, the level of care provided by certain field hospitals, where patients are not admitted, cannot deliver the care that would normally have been given in the original hospital. In addition, due to blocked roads and destruction at the local airport, foreign and inter-island medical assistance was very limited in the first days after Haiyan [17]. The fact that the local population replaced their health needs only during the period with limited accessibility would also explain the catching-up pattern that we see in ODH, where the number of admissions increased to above baseline levels after two weeks. These delays of accessing medical care could also contribute to the high death toll from cyclones, as shown in the systematic review by Doocy et al. [18], due to urgent medical conditions not being properly addressed.

We noticed an overall decrease in the absolute number of patients admitted in the Obstetrics ward and for reasons related to ‘Pregnancy, childbirth and the puerperium’, of which 94% consists of deliveries, after Haiyan compared to baseline (Tables 1 and 2). The evidence about the effects of disasters on pregnancy is mixed: one study suggests that disasters do not influence the gestational period [19], while another suggests that they can cause preterm labour [20]. Therefore, we expected the number of deliveries to either remain stable over time or increase within the study period. The observed decrease could be a matter of concern, because it implies that this difference in deliveries took place outside the hospital environment, although part of the deliveries could have taken place in field hospitals instead. Other studies have shown the impact of disasters on maternal health, such as that exposure to a disaster event impacts maternal mental health [20, 21], which in turn can lead to lower birth weights [22, 23]. Disaster relief should therefore put a stronger focus on ensuring that maternal health care is provided, e.g. by having skilled birth attendants aid in reach-out activities.

Apart from deliveries, Haiyan also affected other types of admissions. In EVRMC, a relative reduction in admissions in the Surgery ward was observed, although this was not seen in ODH (Table 2). This reduction could be due to limited electricity in the hospitals, resulting in postponement of planned but not critical surgical interventions. The increase in orthopaedic admissions in EVRMC is in line with previous studies. A study on the impact of the 2004 Indian Ocean tsunami in Indonesia showed a high proportion of injuries immediately after the event, which decreased after four weeks [4]. Another study showed an increase in trauma admissions in the days following hurricane Isabel in the US [1]. However, more refined data on whether all patients in the Orthopaedic ward in EVRMC were due to injuries would be required to come to a firm conclusion. In ODH, there was a relative increase in both ICD10 groups ‘Certain infectious and parasitic diseases’ and ‘Diseases of the respiratory system’, which mostly consisted of the diseases gastroenteritis and pneumonia, respectively. Samples collected in EVRMC after Haiyan also suggested increased incidence of these conditions [9]. In addition, an admissions-based study in ODH found that the highest burden of paediatric admissions was also due to gastrointestinal illnesses (39.9%) and respiratory tract infections (32.4%) [24]. An outbreak investigation carried out after Haiyan identified consumption of untreated drinking water as the main cause for gastroenteritis [25]. The widespread loss of roofs and protection, especially for vulnerable individuals such as children and the elderly, exposed them to weather conditions and humidity, potentially leading to respiratory infections.

The relationship between lack of roofing and pneumonia has been shown in a study in urban settings in Dhaka, Bangladesh [26]. An increase in pneumonia admissions was also seen after the Tohoku earthquake and tsunami in Japan, especially among elderly aged 65 years or older [3], and after the Great East Japan Earthquake [27]. Hospitals in areas prone to tropical cyclones should thus be prepared for treating larger numbers of patients with gastroenteritis and pneumonia. It is of interest whether this increase in infections and respiratory diseases was also seen in the number of outpatient consultations, since most patients with these conditions are generally not admitted. This is currently under study by the same author group.

We observed some differences in the sex and age patterns between the two hospitals. The absolute decrease in female admissions in ODH after Haiyan compared to the week before Haiyan, and the relative decrease compared to male admissions, were not observed in EVRMC. It may correspond with the decrease in admissions in the ODH Obstetrics ward, although this was not confirmed by our sensitivity analysis without cases admitted to the Obstetrics ward and new-borns. People aged ≥50 years had the highest relative risk of admission in EVRMC after Haiyan compared to baseline, while this was the case for children aged 0–4 years in ODH. Both these age groups are generally observed to be also at risk for increased mortality after tropical cyclones [18].

Our study had some limitations. First, in EVRMC, data on individual diagnoses was missing for a large part of the admissions. We used ward as a proxy for diagnosis, although some wards provide very little information on the type of admissions, e.g. the Medicine ward. Second, due to a high level of destruction in the study area during and after Haiyan, it is not possible to say with certainty how some of the patterns observed in our study can be explained, e.g. direct impact of the typhoon, damage and accessibility to the hospitals, closure of other hospitals, availability of field hospitals or casual seasonal changes. To gain more insight into these underlying patterns, we conducted a qualitative study with hospital personnel, which will be published separately.

### Conclusion

Our study identified several priorities for hospitals in zones at high risk of tropical cyclones. First, because of the observed reduction in patients after the event, accessibility to the hospital should be protected by measures such as removing debris in the direct hospital vicinity and prioritising access for emergency transportation, in order to ensure access for both staff and patients in case of mass emergencies. Second, deliveries will remain a constant through the emergency period and constitute a significant proportion of all hospital care in countries such as the Philippines, where fertility is high. The decrease of admissions in the Obstetrics ward suggests that not all deliveries took place in the hospital setting. Therefore, the capacity to respond to urgent obstetric needs should be foreseen, e.g. by ensuring skilled birth attendants in reach-out activities if needed. Third, the most common conditions for admission after Haiyan were gastroenteritis and pneumonia, confirmed as well by studies elsewhere. Hospitals in high risk zones should therefore be prepared to treat large numbers of patients with these conditions, and this should be incorporated in their disaster plans. Finally, our study brings further evidence to the higher risk of admission faced by children and elderly following an acute event. These findings and recommendations are of particular importance for operational activities after a tropical cyclone.
